# Early postoperative red cell distribution width is associated with prolonged hospital stay and complications after non-cardiac surgery in neonates

**DOI:** 10.7150/ijms.131485

**Published:** 2026-08-11

**Authors:** Jialian Zhao, Miaomiao Hao, Zhongteng Lu, Wenyuan Zhang, Linjie Qiu, Minyue Qian, Chunyi Jin, Binbin Cai, Yang Li, Mei Guo, Kai Zhang, Yue Jin

**Affiliations:** 1 Department of Anesthesiology, Children's Hospital, Zhejiang University School of Medicine, National Clinical Research Center for Children and Adolescents' Health and Diseases, Hangzhou, China; 2 Department of Anesthesiology, the First Affiliated Hospital, Zhejiang University School of Medicine, Hangzhou, China.; 3 Perioperative and Systems Medicine Laboratory, Children's Hospital, Zhejiang University School of Medicine, National Clinical Research Center for Children and Adolescents' Health and Diseases, Hangzhou, China

**Keywords:** Red blood cell distribution width, Neonate, non-cardiac surgery, prolonged hospital stay, 30-day mortality, postoperative complications

## Abstract

**Background:**

Red blood cell distribution width (RDW) has emerged as a potential biomarker associated with adverse outcomes in various diseases. However, its clinical significance in neonatal surgical populations remains unclear. The present study aimed to investigate the association between early postoperative RDW and postoperative outcomes in neonates undergoing non-cardiac surgery.

**Methods:**

This retrospective cohort study included neonatal patients who underwent non-cardiac surgery at a tertiary children's hospital. Patients were stratified into quintiles based on early postoperative RDW levels: Q1 (extremely low), Q2 (low), Q3 (moderate), Q4 (high) and Q5 (extremely high). The primary outcome was postoperative length of hospital stay (LOS). Secondary outcomes included 30-day mortality, duration of postoperative mechanical ventilation and ICU stay, and postoperative complications. Multivariable regression analyses were performed to assess associations. Propensity score matching (PSM) comparing the higher-RDW group with the lower-RDW group was performed to further account for baseline confounding.

**Results:**

A total of 1,014 neonates were included in this study. The RDW quintile cutoffs were defined as follows: Q1 (< 14.5%), Q2 (14.5-15.0%), Q3 (15.1-15.7%), Q4 (15.8-17.1%) and Q5 (> 17.1%). Neonates in the higher quintiles had significantly higher risks for prolonged LOS (Q4: adj. OR 2.516, [95% CI 1.217-5.204]; Q5: adj. OR 5.854, [95% CI 2.606-13.146]; p for Trend < 0.001). After PSM, the increased risk of prolonged LOS remained significant for neonates in the higher-RDW group (Q4-Q5), with an adjusted OR of 3.805 (95% CI 1.998-7.245, p < 0.001) in the matched cohort. When analyzed as a continuous variable, early postoperative RDW remained positively associated with LOS (p = 0.020). This association was consistent across subgroups and sensitivity analyses. For secondary outcomes, the association was also observed in prolonged ICU stay, acute kidney injury and acidosis (all p < 0.05) but there was no difference in 30-day mortality.

**Conclusions:**

Elevated RDW within 24 hours after non-cardiac surgery is significantly associated with prolonged LOS and increased risk of postoperative complications in neonates. RDW may serve as a clinically accessible biomarker for postoperative risk stratification in this population.

## Introduction

Red cell distribution width (RDW) reflects heterogeneity in the size of circulating erythrocytes and is commonly used to evaluate anemia. There is increasing evidence that elevated RDW is associated with adverse outcomes in a wide range of conditions, including cardiovascular diseases 1,2, respiratory diseases 3,4, liver diseases 5, tumors 6, metabolic disorders 7,8. In surgical populations, higher RDW levels have been associated with poor prognosis and prolonged length of hospital stay (LOS) in elder patients 9 and adults undergoing non-cardiac surgery 10.

In pediatric populations, elevated RDW has been associated with increased mortality and prolonged mechanical ventilation time in critically ill children 11,12. RDW also correlates with disease severity and prognosis in pediatric sepsis and has been used as a tool for clinical risk stratification and therapeutic guidance 13. The pathophysiological mechanisms underlying the association between elevated RDW and adverse outcomes are not fully understood, but possible explanations include inflammation, oxidative stress, and impaired erythropoiesis 14. Most existing studies have focused on adult and older pediatric populations; however, the prognostic value of RDW in neonatal surgical patients remains unexplored.

In this retrospective cohort study, we aimed to investigate the association between early postoperative RDW levels and prolonged hospital stay as well as complications in neonatal population undergoing non-cardiac surgery.

## Methods

### Study design and setting

**Figure 1:**
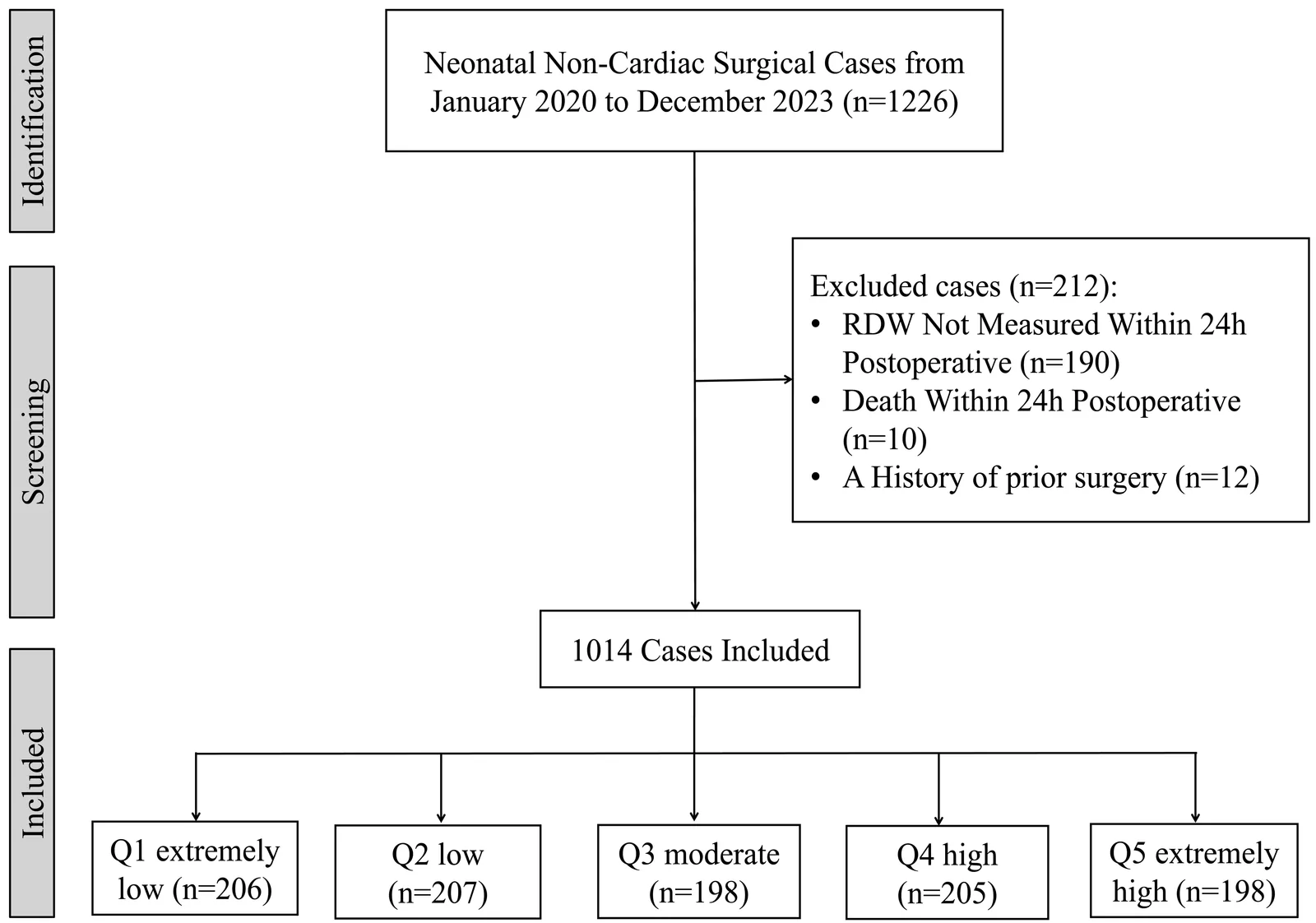
Flow diagram of patient categorization.

The study was approved by the Ethics Committee Board of Children's Hospital, Zhejiang University School of Medicine (2025-IRB-0162-P-01, April 24, 2025) in accordance with the Declaration of Helsinki, and was registered at chictr.org.cn (ChiCTR2500104859, June 25, 2025). A retrospective analysis of data was conducted from January 2020 to December 2023. The study included all neonates who underwent non-cardiac surgery and general anesthesia with endotracheal intubation. Due to the retrospective nature of the study, informed consent was waived by the Ethics Committee. The exclusion criteria were: (1) absence of RDW measurement within 24 hours after surgery, (2) death within 24 hours postoperatively, and (3) A history of prior surgery (Figure 1).

### Data collection and RDW assessment

Patient demographics and perioperative data were extracted from the hospital's clinical information system (Ewell, Hangzhou, China) and anesthesia data management system (Medical System, Suzhou, China). The baseline characteristics included postmenstrual age (PMA) at surgery, gestational age at birth, and chronological age, weight, gender, premature delivery, American Society of Anesthesiologists (ASA) physical status classification, surgery site, preoperative comorbidities and treatments, and laboratory findings. Surgical procedures were categorized into levels 1-4 according to the institutional surgical procedure classification system. Intraoperative variables included the duration of surgery and anesthesia, intraoperative hypotension, hypoxemia, blood loss, blood transfusion, and vasoactive agents. Postoperative complications and outcomes were also recorded. Two researchers independently extracted the data, and a third reviewer resolved any discrepancies.

RDW values were obtained from complete blood count tests performed by laboratory technicians. Early postoperative RDW was defined as the RDW value within 24 hours after surgery 15. RDW at admission was defined as the first RDW measurement during the hospitalization, and preoperative RDW was defined as the most recent RDW value obtained before surgery. Neonates were categorized into five groups according to quintiles of early postoperative RDW: Q1 (extremely low), Q2 (low), Q3 (moderate), Q4 (high) and Q5 (extremely high).

### Outcomes

The primary outcome was the length of postoperative LOS. Secondary outcomes included the postoperative intensive care unit (ICU) stay, duration of postoperative mechanical ventilation, 30-day all-cause mortality, and postoperative complications. Based on the 75th percentile of postoperative LOS, a threshold commonly used to identify patients at increased risk of adverse outcomes 16, prolonged hospitalization was defined as a postoperative LOS exceeding 28 days 17,18. Prolonged ICU stay was defined as a postoperative ICU stay ≥ 10 days, and prolonged postoperative mechanical ventilation was defined as a duration of 36 hours or more.

Postoperative complications include postoperative pulmonary complications (PPCs), acute kidney injury (AKI), acidosis, unplanned reintubation and reoperation. PPCs were defined as the occurrence of a respiratory tract infection, respiratory failure, pleural effusion, atelectasis, pneumothorax, bronchospasm, or aspiration pneumonia 19. AKI was defined according to the modified KDIGO criteria for neonates 20, 21, as the presence of any of the following within 7 days: an increase in serum creatinine of ≥ 0.3 mg/dL within 48 hours; an increase in serum creatinine to ≥ 1.5 times the baseline value; or urine output of < 0.5 mL/kg/hour for ≥ 6 consecutive hours. Acidosis was defined as a pH < 7.35 on blood gas analysis 22 within 7 days postoperatively.

### Statistical analysis

Continuous variables that follow a normal distribution are expressed as the mean **±** standard deviation (Mean ± SD) and were compared using one-way analysis of variance (ANOVA). Non-normally distributed continuous variables are expressed as the median with the interquartile range (IQR) and were compared using the Kruskal-Wallis test. Categorical data were presented as counts and percentages and compared using the chi-squared test or Fisher's exact test with Bonferroni correction when appropriate.

A multivariable logistic regression model was constructed to investigate the association between the quintiles of early postoperative RDW and outcomes. The following covariates were adjusted for: chronological age, weight, gestational age, gender, ASA physical status classification, emergency surgery, surgery level, preoperative respiratory disease, congenital heart disease (CHD), birth asphyxia, preoperative jaundice, preoperative blood transfusion, anesthesia duration, blood loss, intraoperative blood transfusion, vasoactive drugs and preoperative RDW. Sensitivity analyses included propensity score adjustment for above confounding adjustment, as well as best- and worst-case scenario analyses, to assess the influence of the exclusion of early deaths. The results of the model are presented as adjusted odds ratios (adj OR) with their 95% confidence intervals (95% CI). Due to the few events of some outcomes (30-day mortality and AKI), Firth's penalized logistic regression was used for analysis.

To further address potential confounding, we performed propensity score matching (PSM) as sensitivity analysis, while the quintile-based analysis in the full cohort served as the primary analysis. Neonates in Q4-Q5 were categorized as the higher-RDW group, whereas those in Q1-Q3 were categorized as lower-RDW group. Propensity scores were estimated with clinically relevant pre-exposure covariates, including gestational age, birth weight, preoperative and intraoperative transfusion, ASA physical status, emergency surgery, and surgery time. Patients in the higher-RDW group were matched to those in the lower-RDW group at a 1:2 ratio using nearest-neighbor matching with a caliper of 0.25 times the standard deviation of the logit-transformed propensity score. Covariate balance before and after matching was assessed using standardized mean differences (SMDs), with an absolute SMD < 0.1 considered indicative of acceptable balance (supplementary Table 2). In the matched cohort, conditional logistic regression accounting for the matched sets was used to evaluate the association between high RDW and prolonged LOS and other postoperative outcomes.

Receiver operating characteristic (ROC) curves were used to assess the ability of RDW to discriminate between groups at admission, preoperatively, and early postoperatively. The curves were then compared using the DeLong test. Given the overdispersion of LOS, negative binomial regression was applied to investigate the association between early postoperative RDW and LOS. Additionally, restricted cubic spline (RCS) analysis based on the adjusted multivariable model was further performed to illustrate the potential relationship between continuous early postoperative RDW and LOS. Subgroup analyses were conducted according to transfusion, gestational age, ASA physical status, emergency surgery, duration of surgery and preoperative RDW level 23. Kaplan-Meier curves were constructed to compare survival outcomes, and the Log-rank test was used to assess the statistical significance of difference between groups.

Due to the low proportion of missing data, a complete-case analysis was performed. All data were analyzed using SPSS 26.0 (IBM Corp., Armonk, NY, USA), RStudio 4.4.2 (RStudio, Inc., Boston, MA, USA), and GraphPad Prism 9 (GraphPad Software, San Diego, CA, USA). All statistical tests were two-sided, and a p value of less than 0.05 was considered statistically significant.

## Results

### Patient characteristics

From January 2020 to December 2023, 1014 neonates were included in the final analysis (Figure 1). The median chronological age was 6 (2-12) days, and the median weight was 2.9 (2.3-3.4) kg. Abdominal surgery was the most common procedure (813, 80.2%), followed by head and neck surgery (107, 10.6%), and then thoracic surgery (92, 9.1%). The median surgery and anesthesia time were 75 (49.0-105.0) minutes and 130 (96-165) minutes, respectively (Table 1).

Neonates were stratified into five groups: Q1 (< 14.5%, n = 206), Q2 (14.5-15.0%, n = 207), Q3 (15.1-15.7%, n = 198), Q4 (15.8-17.1%, n = 205), and Q5 (> 17.1%, n = 198). Neonates in the higher RDW quintiles were younger and had lower body weights. They also had higher ASA physical status classifications, more preoperative comorbidities, and increased use of perioperative life-support measures (all p < 0.05) (Table 1). Preoperative laboratory parameters are also presented in Table 1.

### RDW and prolonged LOS

**Figure 2:**
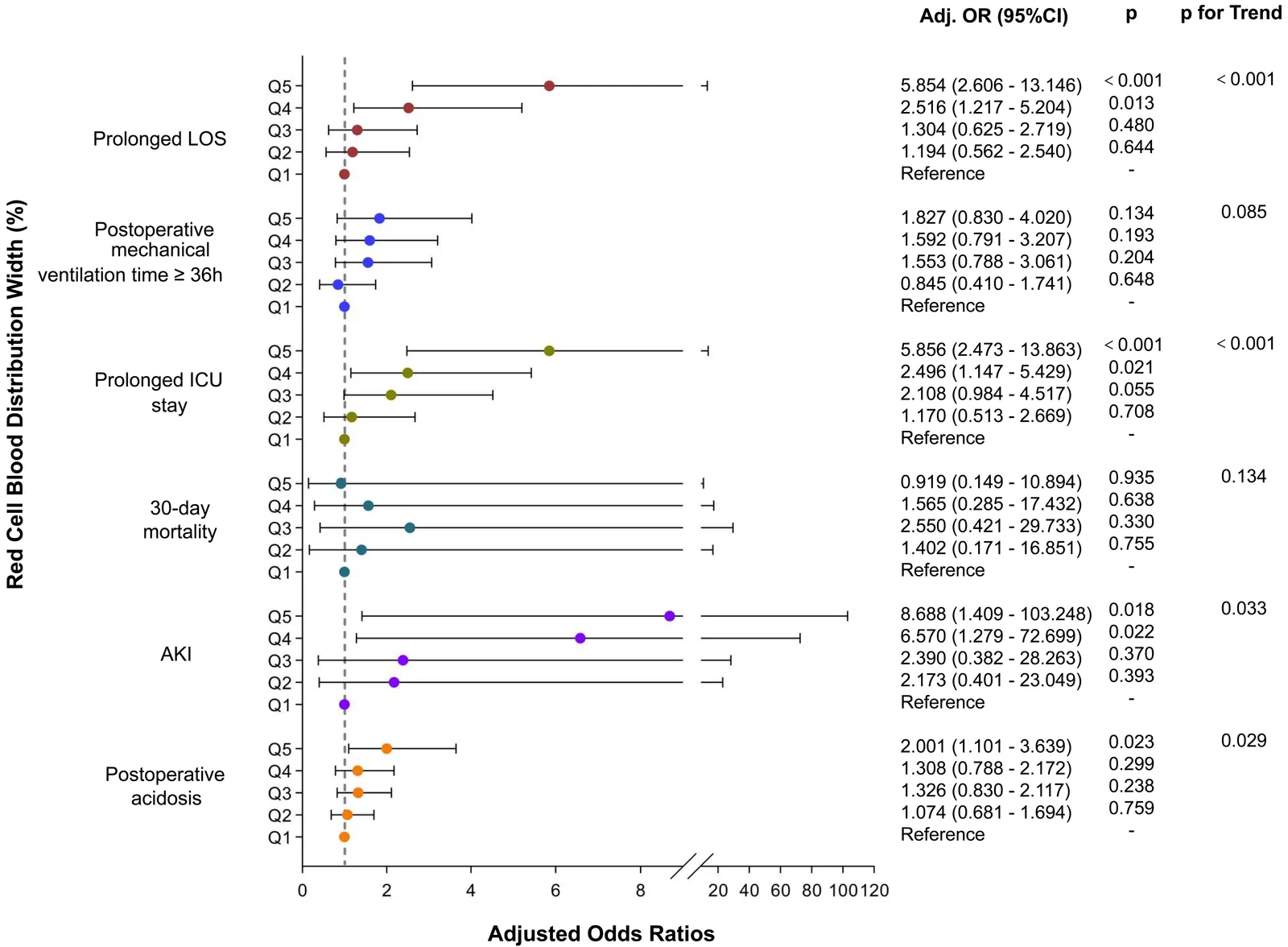
** Association between early postoperative RDW levels and the risk of adverse postoperative outcomes.** Multivariable binary logistic regression was used to analyze the association between early postoperative RDW levels and prolonged LOS, time on mechanical ventilation, ICU stay, 30-day mortality, AKI, and acidosis, after adjusting for potential confounders. Adjusted OR with 95% CI are shown with Q1 as the reference group. AKI: acute kidney injury; CI: confidence interval; ICU: intensive care unit; LOS: length of stay; OR: odds ratio; RDW: red cell distribution width.

The median LOS after surgery for the entire cohort was 14 (8-29) days. Prolonged LOS, defined as a postoperative hospital stay exceeding 28 days, occurred in 254 neonates (25.0%). Postoperative LOS increased significantly with elevated RDW levels. In the extremely high RDW (Q5) group, the median LOS was 37 (19-62) days, and 62.1% experienced prolonged LOS (Table 2). After adjusting for confounders, the high and extremely high early postoperative RDW groups were associated with a prolonged LOS (Q4: adj. OR 2.516, [95% CI 1.217-5.204]; Q5: adj. OR 5.854, [95% CI 2.606-13.146]; p for Trend < 0.001) (Figure 2).

**Figure 3:**
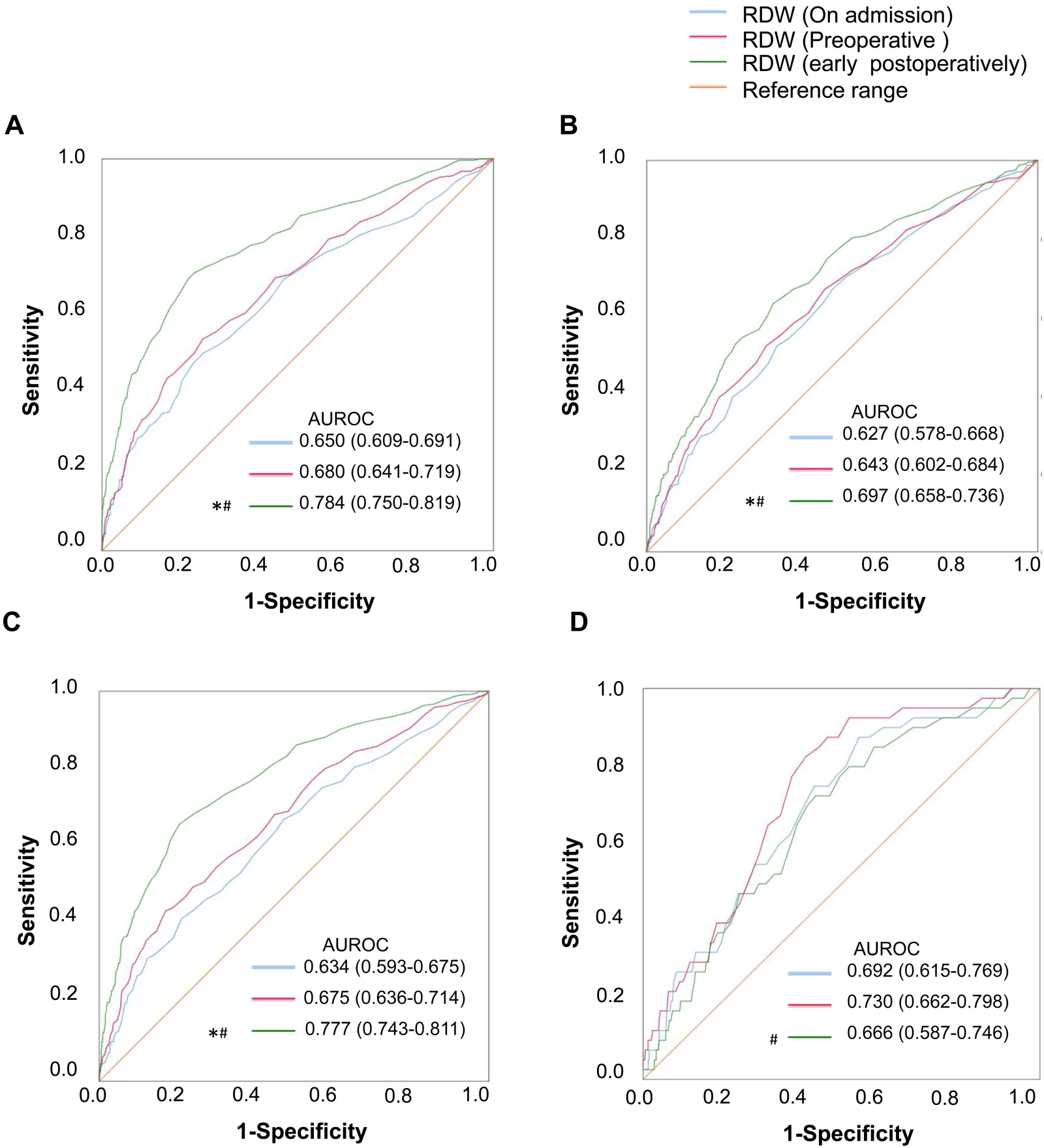
** Receiver operating characteristic curve analysis of early postoperative RDW for discriminating prolonged length of stay** (A) prolonged LOS; (B) postoperative mechanical ventilation time ≥36 hours; (C) prolonged postoperative ICU stay; and (D) 30-day mortality. *, p < 0.05 for the DeLong test of early postoperative RDW AUC vs. preoperative RDW; ^#^, p < 0.05 for the DeLong test of early postoperative RDW AUC vs. admission RDW.

**Figure 4:**
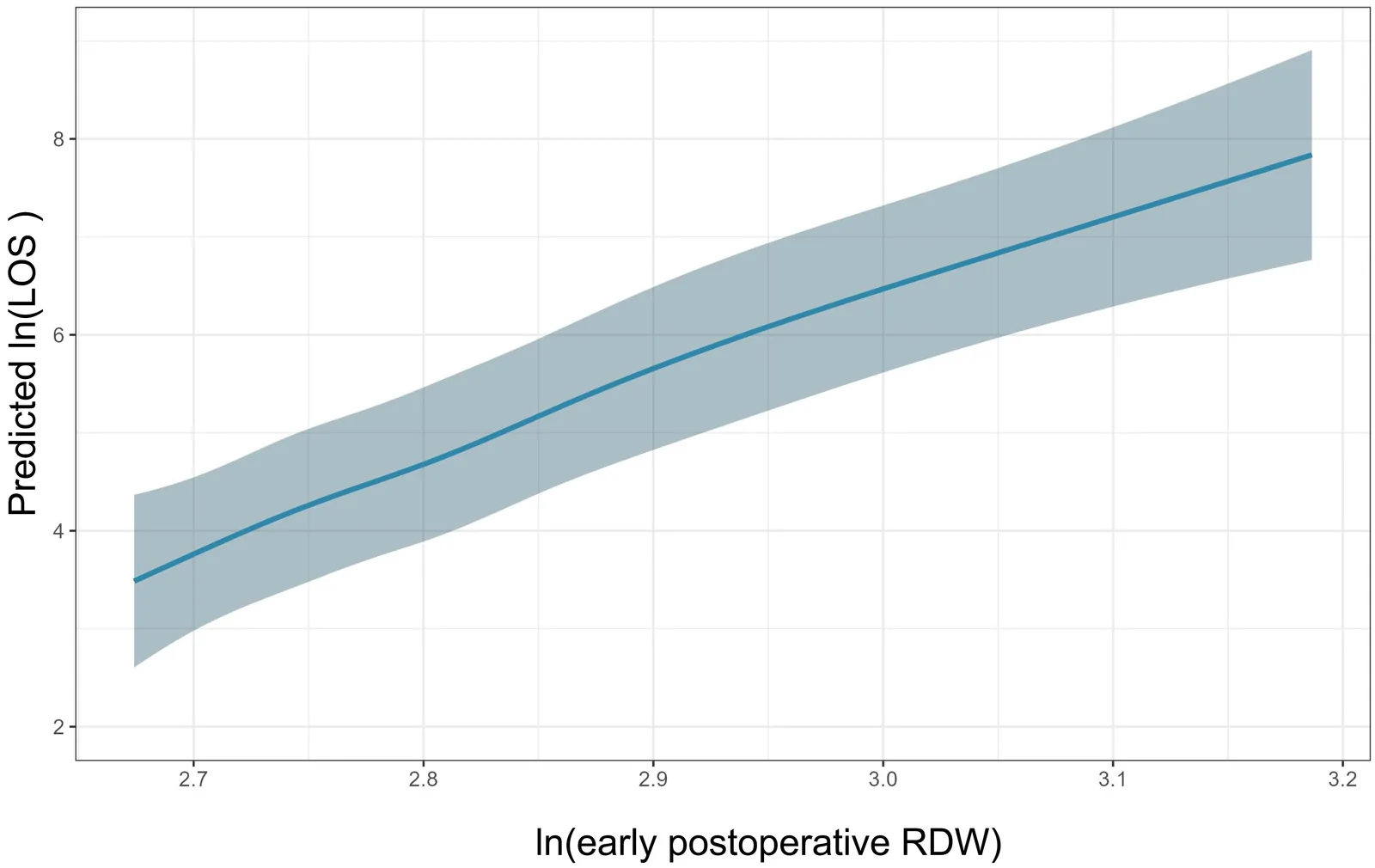
Association between the early postoperative RDW and postoperative length of hospital stay.

ROC curve analysis revealed that early postoperative RDW had good discriminative ability for prolonged LOS, with an area under the curve (AUC) of 0.784 (95% CI: 0.750-0.819; Figure 3A). Early postoperative RDW demonstrated significantly better discrimination for prolonged hospital and ICU stay, as well as prolonged postoperative mechanical ventilation time than RDW at admission or preoperative RDW (all p < 0.05, Figure 3A-C). Restricted cubic spline analysis showed a postoperative association between continuous early postoperative RDW and LOS after adjusting for confounding variables (Figure 4).

### RDW and postoperative complications

In univariate analysis, an elevated RDW level early in the postoperative period was associated with a longer ICU stay, prolonged mechanical ventilation, AKI, blood transfusion, acidosis, unplanned reintubation, unplanned reoperation and 30-day mortality (all p < 0.05) (Table 2). After multivariable adjustment, extremely high postoperative RDW remained an independent risk factor for prolonged ICU stay (≥ 10 days) (Q5: adj. OR 5.856, [95% CI 2.473-13.863], p for trend < 0.001), and the AUC was 0.777 [95% CI 0.743-0.811] (Figure 2 and Figure 3C).

Additionally, neonates in the elevated RDW group had significantly higher risks of AKI (Q4: adj. OR 6.570, [95% CI 1.279 - 72.699]; Q5: adj. OR 8.688, [95% CI 1.409-103.248], p for Trend = 0.033) and acidosis (Q5: adj OR 2.001, [95% CI 1.101-3.639], p for Trend = 0.029) (Figure 2).

### RDW and 30-day mortality

Survival analysis showed significant differences in 30-day survival rates among RDW groups (p < 0.001) (supplementary Figure 1). However, after adjustment, early postoperative RDW was not independently associated with 30-day mortality (Figure 2). In contrast, preoperative RDW was associated with 30-day mortality (AUC = 0.730, [95% CI: 0.662-0.798], p < 0.05) (Figure 3D).

### PSM, sensitivity and subgroup analysis

After propensity score matching, a total of 700 neonates were included in the matched cohort (256 in higher-RDW and 444 in lower-RDW group). In the matched cohort, higher-RDW was significantly associated with prolonged LOS (adjusted OR 3.805, [95% CI 1.998-7.245], p < 0.001), ICU stay ≥ 10 days (adjusted OR 2.192, [95% CI 1.118-4.295], p = 0.022), AKI (adjusted OR 5.868, [95% CI 1.312-26.248], p = 0.021) (Table 3). No significant associations were observed with mechanical ventilation ≥ 36 hours, 30-day mortality, or postoperative acidosis.

**Figure 5:**
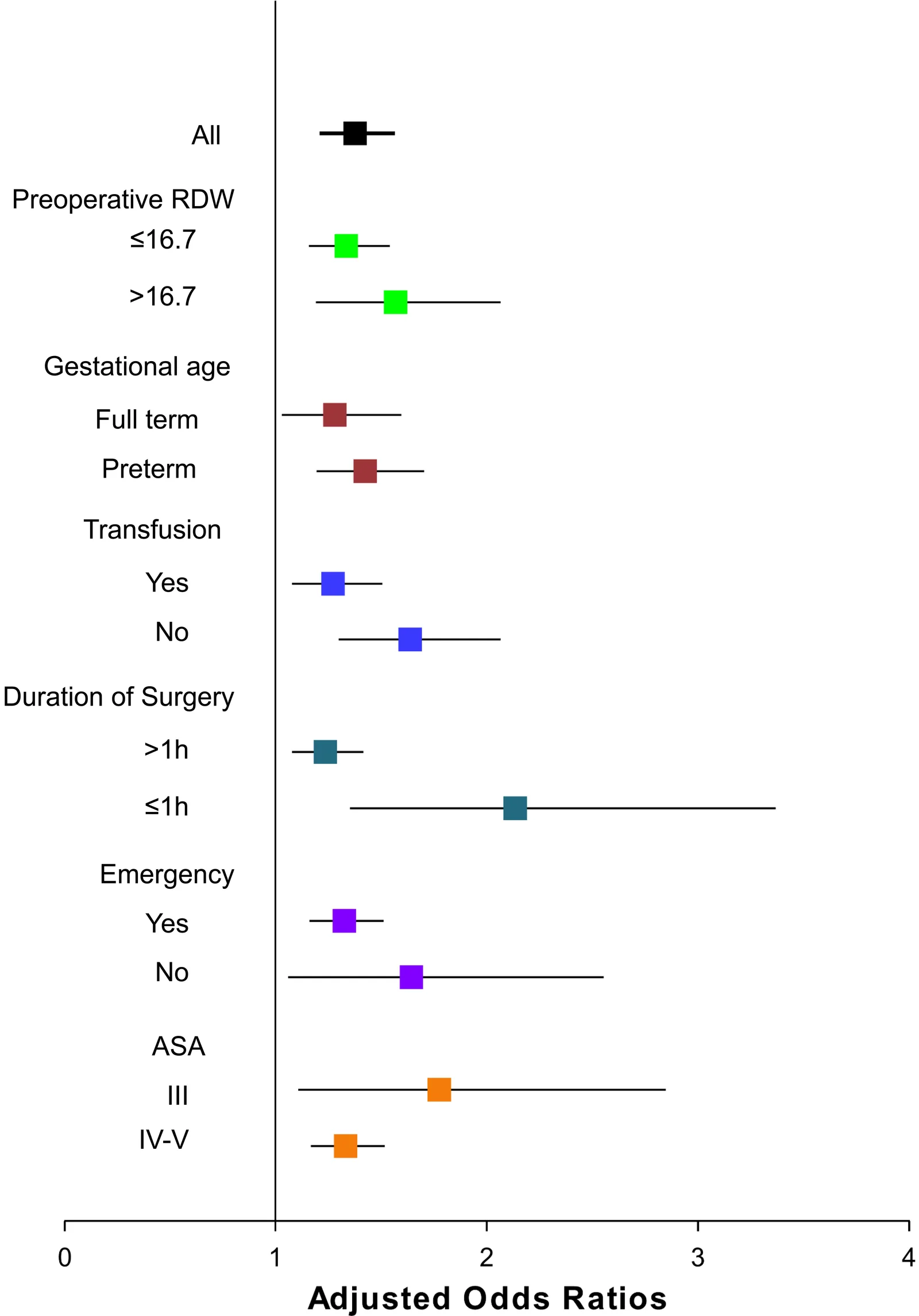
** Subgroup analysis of the association between early postoperative RDW and prolonged hospital stay.** Firth-logistic regression was used to analyze the association between early postoperative RDW levels as continuous variable and prolonged LOS in different subgroups.

After conducting sensitivity analyses that included best- and worst-case scenario analyses, we found that the high and extremely high early postoperative RDW groups were still significantly associated with a prolonged postoperative LOS (supplementary Table 1). Subgroup analysis demonstrated that this association persisted across clinically relevant subgroups stratified by preoperative RDW levels, gestational age, transfusion, surgery duration, emergency surgery, and ASA classification (Figure 5).

## Discussion

In this study, we demonstrated that elevated RDW levels early in the postoperative period, particularly the extremely high quintile, were independently associated with a prolonged LOS as well as postoperative complications, in neonates undergoing non-cardiac surgery. This association remained consistent when the data were stratified by blood transfusion, gestational age, surgical time, and ASA classification. These results suggest that early postoperative RDW may be a readily available and valuable biomarker for identifying neonates at increased risk of adverse outcomes following non-cardiac surgery.

Previous studies have shown that higher levels are associated with increased risks of adverse outcomes and mortality in various patients 24. High RDW is associated with prolonged LOS in adult patients after non-cardiac surgery 10. In elderly patients, RDW values exceeding 16.6% have been linked to higher all-cause mortality 9. In pediatric settings, elevated RDW has been reported to correlate with increased mortality and prolonged mechanical ventilation in critically ill children 11,12. Our findings are consistent with previous evidence. However, as these results reflect unadjusted associations, they should be interpreted with caution. The observed differences are likely to be driven by underlying baseline risk factors rather than RDW itself.

The mechanisms linking elevated RDW to adverse postoperative outcomes in neonates are likely multifactorial. Inflammation during perioperative period induced by surgical stress can impair erythropoietin activity, suppress erythropoiesis, alter red blood cell membrane deformability, and increase the production of ineffective erythrocytes 25-28. This results in greater anisocytosis and elevated RDW levels. Additionally, perioperative oxidative stress, triggered by blood loss, ischemia-reperfusion injury, and hypoxia, may shorten erythrocyte lifespan and accelerate erythrocyte senescence, contributing further to increased RDW levels 26,29. Furthermore, oxidative stress-mediated activation of inflammatory pathways and complement components can promote tissue injury and organ dysfunction and accelerate erythrocyte senescence and clearance, thereby linking elevated RDW to worse postoperative outcomes and mortality 30,31.

We also observed a significant association between elevated RDW levels and postoperative AKI. This finding is consistent with prior studies that identified RDW as an independent risk factor for AKI 32. The systemic inflammation and oxidative stress associated with elevated RDW may contribute to renal tubular epithelial injury and renal tissue hypoxia, thereby exacerbating kidney dysfunction 33,34. Considering the strong link between AKI and mortality in neonates, the association between RDW and AKI further highlights the prognostic significance of this hematologic parameter.

From a clinical perspective, RDW is an inexpensive, readily available laboratory index that does not require additional testing or cost. Incorporating RDW measurements into the routine assessment of neonates before and after surgery may facilitate the early identification of high-risk patients, enabling closer monitoring and targeted management. While specific interventions targeting RDW itself have yet to be established, elevated RDW levels may prompt clinicians to consider strategies that optimize anemia, inflammation, nutritional status, and overall physiological reserve before and after surgery.

There are several limitations of this study that should be acknowledged. First, it was a single-center retrospective analysis, which may limit the generalizability of the findings. Second, RDW is influenced by multiple factors, including anemia, blood transfusions, hemorrhage, and inflammatory conditions. Although we adjusted for known confounders and performed sensitivity analyses, residual unmeasured confounding factors may still exist. Third, RDW was only assessed at three time points, which precluded evaluation of dynamic changes in RDW and their temporal relationship with postoperative outcomes. Although preoperative RDW was included as a covariate in our analyses, residual confounding and potential effect modification by baseline RDW cannot be completely excluded. Finally, neonatal AKI was defined using modified KDIGO criteria. The lack of universally accepted definition of neonatal AKI may have introduced bias into our results.

## Conclusion

In summary, elevated red blood cell distribution width level early in the postoperative period is independently associated with a prolonged hospital stay and postoperative complications in neonates undergoing non-cardiac surgery. These results imply that RDW could be a useful and accessible tool for assessing perioperative risk in neonatal surgical care.

## Supplementary Material

Supplementary figures and tables.

## Figures and Tables

**Table 1:** Baseline and perioperative characteristics according to early postoperative RDW quintiles.

	Total (n = 1014)	Quintile of RDW					p
		Q1 (< 14.5%) (n = 206)	Q2 (14.5-15.0%) (n = 207)	Q3 (15.1-15.7%) (n = 198)	Q4 (15.8-17.1%) (n = 205)	Q5 (> 17.1%) (n = 198)	
Postmenstrual age at surgery, week	38 (36-41)	40 (39-42)	39 (38-41)	39 (37-40)	38 (36-40)	35 (32-39)	< 0.001
Gestational age, week	38 (35-39)	38 (37-39)	38 (37-39)	38 (36-39)	37 (35-39)	33 (30-37)	< 0.001
Chronological age, day	6 (2-12)	8 (4-15)	5 (2-11)	5 (2-9)	4 (2-9)	7 (3-15)	< 0.001
Weight, kg	2.9 (2.3-3.4)	3.1 (2.7-3.5)	3.1 (2.7-3.5)	2.9 (2.5-3.4)	2.9 (2.1-3.3)	2.0 (1.5-2.9)	< 0.001
Gender, male	566 (55.8)	111 (53.9)	109 (52.7)	113 (57.1)	123 (60.0)	110 (55.6)	0.603
Prematurity	343 (33.8)	31 (15.0)	30 (14.5)	62 (31.3)	90 (43.9)	130 (65.7)	< 0.001
ASA physical status							< 0.001
Ⅲ	399 (39.3)	103 (50.0)	101 (48.8)	98 (49.5)	71 (34.6)	26 (13.1)	
Ⅳ	431 (42.5)	88 (42.7)	96 (46.4)	78 (39.4)	86 (42.0)	83 (41.9)	
V	184 (18.1)	15 (7.3)	10 (4.8)	22 (11.1)	48 (23.4)	89 (44.9)	
Emergency surgery	787 (77.6)	133 (64.6)	155 (74.9)	147 (74.2)	170 (82.9)	182 (91.9)	< 0.001
Surgery site							0.207
Abdomen	813 (80.2)	157 (76.2)	169 (81.6)	161 (81.3)	167 (81.5)	159 (80.3)	
Head and neck	107 (10.6)	28 (13.6)	16 (7.7)	19 (9.6)	16 (7.8)	28 (14.1)	
Thoracic	92 (9.1)	21 (10.2)	22 (10.6)	18 (9.1)	21 (10.2)	10 (5.1)	
Limbs	2 (0.2)	0	0	0	1 (0.5)	1 (0.5)	
Surgery Level							< 0.001
1	2 (0.2)	2 (1.0)	0	0	0	0	
2	92 (9.1)	20 (9.7)	27 (13.0)	21 (10.6)	16(7.8)	8 (4.0)	
3	293 (28.9)	31 (15.0)	33 (15.9)	50 (25.3)	71 (34.6)	108 (54.5)	
4	627 (61.8)	153 (74.3)	147 (71.0)	127 (64.1)	118 (57.6)	82 (41.4)	
Preoperative disease							
Respiratory disease	306 (30.2)	32 (15.5)	34 (16.4)	48 (24.2)	85 (41.5)	107 (54.0)	< 0.001
CHD	786 (77.5)	159 (77.2)	148 (71.5)	161 (81.3)	161 (78.5)	157 (79.3)	0.169
Asphyxia	90 (8.9)	10 (4.9)	4 (1.9)	12 (6.1)	23 (11.2)	41 (20.7)	< 0.001
Jaundice	534 (52.7)	99 (48.1)	97 (46.9)	95 (48.0)	117 (57.1)	126 (63.6)	0.002
Anemia	154 (15.2)	16 (7.8)	18 (8.7)	19 (9.6)	33 (16.1)	68 (34.3)	< 0.001
Preoperative therapy							
Machine ventilation	223 (22.0)	22 (10.7)	20 (9.7)	30 (15.2)	57 (27.8)	94 (47.5)	< 0.001
Vasoactive drug	73 (7.2)	5 (2.4)	5 (2.4)	8 (4.0)	21 (10.2)	34 (17.2)	< 0.001
Transfusion	104 (10.3)	9 (4.4)	9 (4.3)	9 (4.5)	32 (15.6)	45 (22.7)	< 0.001
Preoperative laboratory results							
Hemoglobin, g/L	152 (126-177)	150 (133-174)	161 (138-182)	158 (139-180)	158 (128-183)	123 (104-156)	< 0.001
RBC, ×10¹²/L	4.1 (3.5-4.7)	4.3 (3.7-4.7)	4.4 (3.8-4.9)	4.2 (3.7-4.8)	4.3 (3.6-5.0)	3.7 (3.0-4.4)	< 0.001
WBC, ×10⁹/L	11.4 (8.7-16.1)	11.8 (9.1-16.7)	11.9 (9.3-16.7)	11.8 (8.8-15.7)	11.6 (8.4-15.9)	10.4 (6.7-14.7)	< 0.001
NEU, ×10⁹/L	7.0 (4.0-10.9)	6.2 (4.2-11.2)	7.8 (4.7-11.9)	7.3 (4.1-10.8)	7.7 (3.9-11.1)	5.9 (2.9-9.2)	< 0.001
MCV, fL	103.3 (99.3-107.3)	102.4 (98.3-105.5)	103.4 (99.4-107.1)	104.2 (99.7-106.9)	103.5 (100.2-107.7)	105 (98.5-110.1)	0.003
Albumin, g/L	34.0 (30.7-36.5)	34.8 (32.5-37.5)	34.9 (32.4-37.4)	34.1 (31.6-36.1)	33.3 (30.2-36.1)	30.3 (27.5-34.1)	< 0.001
PT, s	13.0 (11.8-14.3)	12.2 (11.4-13.4)	12.7 (11.7-14.1)	13.3 (12.0-14.3)	13.6 (12.2-15.1)	13.4 (12.0-15.0)	< 0.001
Lactate, mmol/L	2.3 (1.6-3.5)	2.3 (1.6-3.4)	2.5 (1.7-3.7)	2.3(1.5-3.3)	2.5 (1.6-3.8)	2.1 (1.5-3.1)	0.011
Duration of surgery, min	75 (49-105)	71 (40-100)	74 (47-110)	75 (44-102)	75 (55-102)	81 (58-114)	0.067
Duration of anesthesia, min	130 (96-165)	123 (90-157)	127 (95-168)	125 (90-164)	133 (100-156)	140 (112-170)	0.007
Blood loss, ml	2 (1-5)	2 (1-4)	2 (1-5)	2 (1-5)	2 (2-5)	5 (2-5)	< 0.001
Intraoperative hypoxemia	112 (11.0)	19 (9.2)	13 (6.3)	18 (9.1)	27 (13.2)	35 (17.7)	0.003
Intraoperative hypotension	452 (44.6)	67 (32.5)	78 (37.7)	87 (43.9)	117 (57.1)	103 (52.0)	< 0.001
Intraoperative blood transfusion	169 (16.7)	12 (5.8)	12 (5.8)	13 (6.6)	41 (20.0)	91 (46.0)	< 0.001
Intraoperative vasoactive drugs	239 (23.6)	22 (10.7)	31 (15.0)	41 (20.7)	69 (33.7)	76 (38.4)	< 0.001

Data are presented as median (quartile) or n (%). ASA: American Society of Anesthesiologists; CHD: Congenital heart disease; MCV: Mean corpuscular volume; NEU: Neutrophil count; PT: Prothrombin time; RBC: Red blood cell count; RDW: Red blood cell distribution width; WBC: White blood cell count.

**Table 2:** Postoperative outcomes according to early postoperative RDW quintiles.

	Total (n = 1014)	Quintile of RDW					p
		Q1 (< 14.5%) (n = 206)	Q2 (14.5-15.0%) (n = 207)	Q3 (15.1-15.7%) (n = 198)	Q4 (15.8-17.1%) (n = 205)	Q5 (> 17.1%) (n = 198)	
Postoperative LOS, days	14 (8-29)	10 (6-16)	11 (7-17)	12 (8-21)	17 (11-36)	37 (19-62)	< 0.001
Postoperative LOS > 28 days	254 (25.0)	17 (8.3)	20 (9.7)	31 (15.7)	63 (30.7)	123 (62.1)	< 0.001
30-day mortality	40 (3.9)	3 (1.5)	3 (1.4)	8 (4.0)	11 (5.4)	15 (7.6)	0.005
Postoperative mechanical ventilation time, hours	15 (8-29)	9 (6-16)	10 (6-18)	14 (8-23)	19 (10-49)	27 (15-73)	< 0.001
Postoperative ICU stay, days	2 (1-9)	1 (1-2)	1 (1-3)	2 (1-6)	4 (1-12)	18 (2-43)	< 0.001
PPCs	378 (37.3)	66 (32.0)	75 (36.2)	74 (37.4)	82 (40.0)	81 (40.9)	0.630
AKI	38 (3.7)	3 (1.5)	4 (1.9)	4 (2.0)	15 (7.3)	12 (6.1)	0.002
Postoperative blood transfusion	422 (41.6)	50 (24.3)	47 (22.7)	66 (33.3)	104 (50.7)	155 (78.3)	< 0.001
Postoperative acidosis	362 (35.7)	54 (26.2)	59 (28.5)	67 (33.8)	76 (37.1)	106 (53.5)	< 0.001
Unplanned reintubation	32 (3.2)	3 (1.5)	2 (1.0)	3 (1.5)	7 (3.4)	17 (8.6)	< 0.001
Unplanned reoperation	31 (3.1)	1 (0.5)	2 (1.0)	6 (3.0)	9 (4.4)	13 (6.6)	0.002

Data are presented as median IQR or n (%). AKI: acute kidney injury; ICU: intensive care unit; LOS: length of stay; PPCs: postoperative pulmonary complications; RDW: Red blood cell distribution width.

**Table 3:** Early postoperative RDW levels and the risk of adverse postoperative outcomes after propensity score matching in higher-RDW (Q4-Q5) groups compared with lower-RDW (Q1-Q3) group.

	Unadjusted OR (95% CI)	Unadjusted p value	Adjusted OR (95% CI)	Adjusted p value
Prolonged LOS	3.170 (2.041-4.922)	< 0.001	3.805 (1.998-7.245)	< 0.001
Postoperative mechanical ventilation time ≥ 36h	1.792 (1.173-2.737)	0.007	1.779 (0.986-3.207)	0.056
Postoperative ICU stay duration ≥ 10 days	2.083 (1.338-3.242)	0.001	2.192 (1.118-4.295)	0.022
30-day mortality	0.943 (0.414-2.147)	0.889	0.935 (0.128-6.833)	0.947
AKI	4.721 (1.973-11.298)	< 0.001	5.868 (1.312-26.248)	0.021
Postoperative acidosis	1.322 (0.954-1.833)	0.094	1.191 (0.802-1.770)	0.385

CI: confidence interval; ICU: intensive care unit; LOS: length of stay; OR: odds ratio; RDW: red cell distribution width.

## Abbreviations

Alb: albumin
AKI: acute kidney injury
ASA: American Society of Anesthesiologists
CHD: congenital heart disease
CI: confidence intervals
ICU: intensive care unit
IQR: interquartile range
LOS: length of stay
MCV: mean corpuscular volume
NEU: neutrophil count
PMA: postmenstrual age
PPCs: postoperative pulmonary complications
PSM: propensity score matching
PT: prothrombin time
RBC: red blood cell count
RCS: restricted cubic spline
RDW: red blood cell distribution width
ROC: receiver operating characteristic
SMDs: standardized mean differences
WBC: white blood cell count
