# Supplementary material for: Early postoperative red cell distribution width is associated with prolonged hospital stay and complications after non-cardiac surgery in neonates
Source: Int J Med Sci. 2026 Aug 11;23(9):3000–9. doi: 10.7150/ijms.131485 (PMC13539924; doi:10.7150/ijms.131485)

Supplementary Table 1. Worst/Best-case scenario analyses on early postoperative RDW levels and the risk of adverse outcomes.

worst-case scenario analyses		p value	OR	95% CI lower limit	95% CI upper limit
Prolonged LOS	Q1	reference	-	-	-
	Q2	0.479	1.307	0.623	2.740
	Q3	0.417	1.353	0.653	2.803
	Q4	0.015	2.446	1.190	5.030
	Q5	<0.001	5.450	2.437	12.186
Postoperative mechanical ventilation time ≥36h	Q1	reference	-	-	-
	Q2	0.797	0.911	0.446	1.857
	Q3	0.187	1.575	0.802	3.093
	Q4	0.188	1.596	0.795	3.202
	Q5	0.151	1.779	0.810	3.904
Postoperative ICU stay duration ≥10 days	Q1	reference	-	-	-
	Q2	0.509	1.314	0.584	2.955
	Q3	0.047	2.162	1.011	4.625
	Q4	0.020	2.504	1.153	5.435
	Q5	<0.001	5.623	2.376	13.312
30-day mortality	Q1	reference	-	-	-
	Q2	0.314	2.781	0.380	20.356
	Q3	0.137	4.196	0.634	27.759
	Q4	0.393	2.275	0.346	14.982
	Q5	0.753	1.368	0.194	9.673
AKI	Q1	reference	-	-	-
	Q2	0.280	2.663	0.450	15.753
	Q3	0.278	2.735	0.444	16.832
	Q4	0.011	9.335	1.685	51.707
	Q5	0.025	8.107	1.305	50.369
Postoperative acidosis	Q1	reference	-	-	-
	Q2	0.688	1.098	0.697	1.729
	Q3	0.213	1.346	0.843	2.147
	Q4	0.294	1.31	0.791	2.169
	Q5	0.027	1.958	1.078	3.555
**Best-case scenario analyses**		**p value**	**OR**	**95% CI lower limit**	**95% CI upper limit**
Prolonged LOS	Q1	reference	-	-	-
	Q2	0.617	1.212	0.571	2.572
	Q3	0.433	1.340	0.645	2.784
	Q4	0.010	2.589	1.258	5.329
	Q5	<0.001	6.226	2.786	13.912

Postoperative mechanical ventilation time ≥ 36h	Q1	reference	-	-	-
	Q2	0.651	0.847	0.413	1.737
	Q3	0.201	1.551	0.792	3.039
	Q4	0.157	1.649	0.825	3.296
	Q5	0.078	2.014	0.925	4.384
Postoperative ICU stay duration ≥ 10 days	Q1	reference	-	-	-
	Q2	0.721	1.161	0.512	2.632
	Q3	0.054	2.099	0.987	4.466
	Q4	0.016	2.58	1.197	5.564
	Q5	<0.001	6.253	2.67	14.641
30-day mortality	Q1	reference	-	-	-
	Q2	0.314	2.781	0.380	20.356
	Q3	0.137	4.196	0.634	27.759
	Q4	0.393	2.275	0.346	14.982
	Q5	0.753	1.368	0.194	9.673
AKI	Q1	reference	-	-	-
	Q2	0.540	1.787	0.279	11.451
	Q3	0.468	2.012	0.304	13.323
	Q4	0.012	9.342	1.637	53.320
	Q5	0.012	11.434	1.697	77.029
Postoperative acidosis	Q1	reference	-	-	-
	Q2	0.739	1.080	0.686	1.700
	Q3	0.195	1.360	0.854	2.166
	Q4	0.235	1.355	0.821	2.237
	Q5	0.010	2.181	1.208	3.936

Supplementary Table 2. Covariate balance before and after PSM between postoperative lower-RDW vs higher-RDW groups.

Covariate	Before matching (n=1014)			After matching (n=700)			Balanced
	Lower-RDW (n=611)	Higher-RDW (n=403)	SMD	Lower-RDW (n=444)	Higher-RDW (n=256)	SMD	
Gestational age	38 (37-39)	36 (31-38)	-0.692	38 (36-39)	37 (35,39)	-0.082	Yes
Birth weight	3.0 (2.68-3.46)	2.5 (1.7-3.1)	-0.626	3.0 (2.6-3.4)	2.9 (2.2-3.34)	-0.077	Yes
Transfusion	55 (9%)	172 (42.7%)	0.337	55 (12.4%)	53 (20.7%)	0.006	Yes
Duration of surgery	73 (44-103.5)	80 (55-105.5)	0.135	75 (48-105)	74 (52-100)	-0.024	Yes
ASA physical status							
3	302 (49.4%)	97 (24.1%)	-0.254	184 (41.4%)	92 (35.9%)	-0.012	Yes
4	262 (42.9%)	169 (41.9%)	-0.009	215 (48.4%)	118 (46.1%)	-0.033	Yes
5	47 (7.7%)	137 (34%)	0.263	45 (10.1%)	46 (18%)	0.045	Yes
Emergency surgery	435 (71.2%)	352 (87.3%)	0.162	357 (80.4%)	214 (83.6%)	0.021	Yes

Supplementary Figure 1.Survival curves for 30-day mortality after non-cardiac surgery among different RDW quintiles. Differences across all quintiles were statistically significant (log-rank test, p < 0.001).

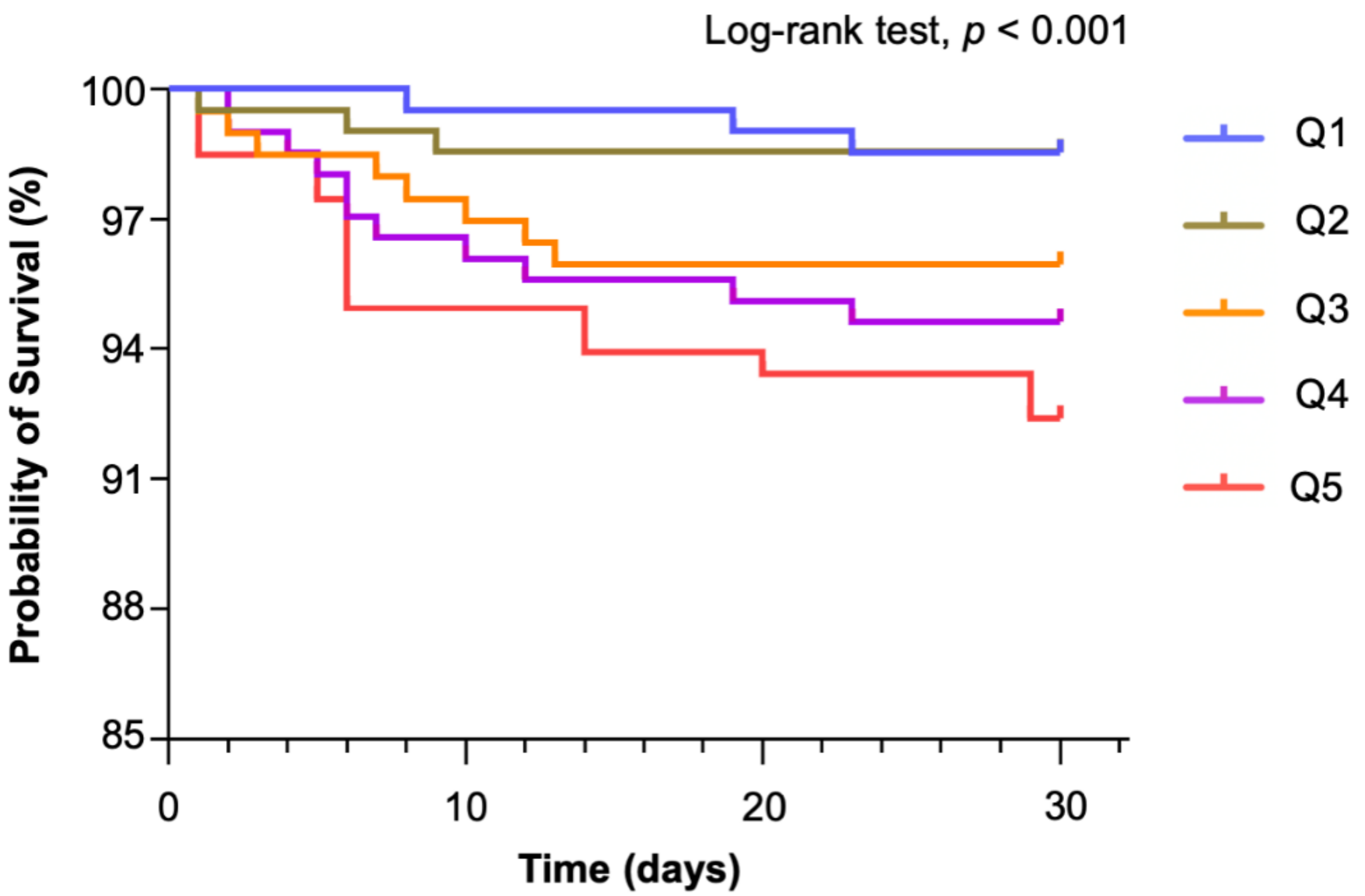

Supplement: Supplementary file 1 [file ijmsv23p3000s1.pdf]
